# Multifunctional Hybrid Fiber Composites for Energy Transfer in Future Electric Vehicles

**DOI:** 10.3390/ma15186257

**Published:** 2022-09-08

**Authors:** Till Julian Adam, Peter Wierach, Pierre Mertiny

**Affiliations:** 1 German Aerospace Center (DLR e. V.), Institute of Composite Structures and Adaptive Systems, Department of Multifunctional Materials, Lilienthalplatz 7, 38108 Braunschweig, Germany; 2 Department of Mechanical Engineering, University of Alberta, Edmonton, AB T6G 1H9, Canada

**Keywords:** multifunctional materials, fiber-reinforced plastics, aluminum fibers, fine wire, structural energy transfer, integrated cables, combined electrical-mechanical loads, electro-mobility, electric aircraft

## Abstract

Reducing the weight of electric conductors is an important task in the design of future electric air and ground vehicles. Fully electric aircraft, where high electric energies have to be distributed over significant distances, are a prime example. Multifunctional composite materials with both adequate structural and electrical properties are a promising approach to substituting conventional monofunctional components and achieving considerable mass reductions. In this paper, a hybrid multifunctional glass-fiber-reinforced composite containing quasi-endless aluminum fibers with a diameter of 45 μm is proposed for electric energy transfer. In addition to characterizing the material’s behavior under static and fatigue loads, combined electrical-mechanical tests are conducted to prove the material’s capability of carrying electric current. Light microscopy, thermal imaging and potentiometry-based resistance characterization are used to investigate the damage behavior. It is found that a volume fraction of about 10% work-hardened aluminum fibers does not affect the static fiber-parallel material properties significantly. Under transverse loading, however, the tensile strength is found to decrease by 17% due to the weak bonding of the aluminum fibers. The fiber-parallel fatigue strength of the multifunctional laminate containing work-hardened aluminum fibers is comparable to that of the reference material. In contrast, the integration of soft-annealed aluminum fibers decreases the tensile strength (−10%) and fatigue life (−21%). Concerning the electrical properties, electrical resistance is nearly unchanged until specimen rupture under quasi-static tensile loads, whereas under cyclic loading, it increases up to 60% within the last third of the fatigue life. Furthermore, the material’s capability of carrying currents up to 0.32 A/mm^2^ (current density of 4.5 A/mm^2^ in the aluminum phase) is proven. Under combined electrical-mechanical loads, a notable reduction in the fatigue life (−20%) is found at low fatigue loads, which is attributed to ohmic specimen heating. To the best knowledge of the authors, this is the first study on the electrical and mechanical material properties and damage behavior of glass-fiber-reinforced composites containing aluminum fibers tested under combined electrical-mechanical loads.

## 1. Introduction

**Figure 1:**
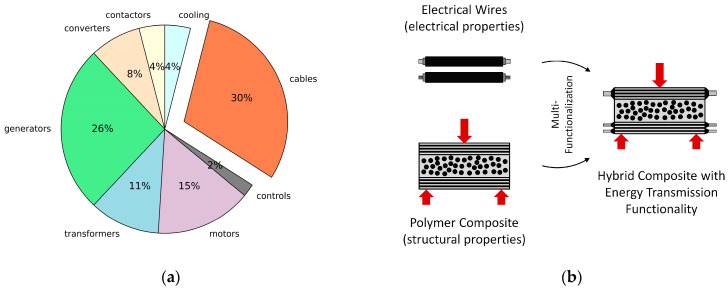
(**a**) Electrical system weight of an electric 300-seater aircraft, according to Gohardani et al. [8]; (**b**) concept of multifunctional electrically conductive and load-bearing (red arrows) composites for transferring energy from storage to electrical consumers.

With electric propulsion evolving into a key technology for future transport vehicles, extensive efforts to decrease the electrical system weight are underway, especially in the aerospace sector, where mass is a main design driver. Reaching beyond optimization on the component level, holistic approaches, such as the concept of multifunctionality, have gained increasing attention within the last two decades. Multifunctional energy storing composite materials, for example, are seen as an opportunity to realize competitive electric road vehicles and energy-saving future aircrafts [1,2,3]. In addition to energy storage, the transfer of high electric energies is also an issue. An illustrative example is given by Warwick [4] for the case of a fully electric regional jet: distributing 1 MW over a distance of 46 m requires about 900 kg of cable when the system voltage is 540 V. The primary approach to decrease the cable mass, of course, is to increase the operating voltage. At a voltage of 2000 V, the cable mass can be reduced to 200 kg [4]. Although this approach might be viable for the main power transmission lines of electric propulsion systems, it does not apply to the tremendous amount of electric wiring of today’s state-of-the-art airliners. With metallic fuselage structures replaced by carbon-fiber-reinforced composite structures, the so-called Electrical Structure Network (ESN), a conductive network of more than 6000 (A350 XWB) conductive components, became necessary in order to ensure the proper functioning of the electrical aircraft systems [5,6,7]. For a 300-seat electric aircraft, Gohardani et al. [8] estimate the total mass of the cables to be 30% of the mass of the electrical system (Figure 1a).

By rethinking the use of conventional electric cables, significant mass savings may be realized by using multifunctional conductive structural materials, as illustrated in Figure 1b. Electric conductive carbon-fiber-reinforced composites based on nano-scaled matrix modifications or coated fibers have been examined comprehensively for different types of application (power transfer, lightning protection, damage monitoring) [8,9,10,11,12,13]. However, with carbon having a rather high electrical resistivity of about $\;3.5\times 10^{-5}\;\Omega m$, and the polymer matrix being an insulator $\left(\gg 10^{10}\;\Omega m\right)$, the resulting composite conductivities are much lower than those of metallic conductors, such as copper ($1.68\times 10^{-8}\;\Omega m$).

An obvious approach that may be used to significantly improve conductivity is to use hybrid composite materials containing a metallic phase. Fiber metal laminates (FMLs) consist of thin metal layers bonded with layers of fiber-reinforced polymer composites. Various types of FMLs, such as the well-known glass-reinforced aluminum (GLARE) [14,15,16] or laminates of carbon-fiber-reinforced plastics with sheets of steel [17] or titanium [18], have been developed. In the first place, these materials are known for their exceptional damage tolerance behavior, corrosion and fire resistance. However, electrical conductivity only plays a subordinate role. The large-area integration of copper mesh and expanded foils is the standard lightning strike protection strategy for today’s composite aircrafts [19]. A more targeted approach to supplying electrical consumers is to embed conductive tracks of copper foil/mesh [20,21], steel foil [22,23] or steel wire [24]. These delimited meso-scaled metallic interlayers, however, have been observed to weaken the composite locally [23,25].

The micro-scaled integration of very thin continuous metal fibers (superfine wire, diameter <100 µm) into single plies is a more elaborate approach. According to Hannemann et al. [26], a rather high-volume fraction of 20% steel fibers is required to provide an eightfold increase in conductivity compared to conventional CFRPs. So-called SFRPs (steel-fiber-reinforced plastics), however, suffer from significant mass increases (specific mass of steel: $7.8\;g/{\mathrm{cm}}^{3}$, carbon: $1.6\;g/{\mathrm{cm}}^{3}$). This also holds true for most other metallic conductors.

To avoid undesired density increases associated with most metal conductors, a hybrid composite containing glass fibers and aluminum fibers is proposed and investigated in this study. In the first step, several approaches to metal fiber integration are presented and discussed. The material configuration chosen in this study is manufactured by winding unidirectional aluminum fiber prepregs, which are then combined with a unidirectional glass fiber plain weave fabric. In addition to characterizing the mechanical properties of the reference GFRP and three configurations with aluminum fibers, the material performance is also assessed for combined electrical and mechanical loads, applying different measurement and monitoring techniques such as microscopy, potentiometry and thermography.

The main objectives of this work are to:

- Assess the mechanical performance of the multifunctional material for static and fatigue loads;
- Prove that the material can carry technically relevant electrical currents without the mechanical properties being affected;
- Gain an understanding of the damage mechanisms under mechanical and combined electrical/mechanical loads;
- Discuss the material’s capabilities and potential for technical applications.

## 2. Approach: Aluminum-Fiber-Glass-Fiber-Reinforced Plastic (AlFGFRP)

Reinforcing conventional epoxy matrix materials with both glass and aluminum fibers is proposed in order to obtain an electrically conductive light-weight material, referred to as AlFGFRP. Although the combination of GFRP and fibrous aluminum is challenging in many regards (e.g. fabrication, providing interfacial bonding, compatibility of the constituents, the effect of non-elastic material deformation and the difficult electrical contacting), it is promising in view of the mechanical compatibility of the constituents and electrical properties. In contrast to the number of studies investigating SFRPs (containing fibrous steel), hardly any publications on hybrid aluminum fiber composites can be found in literature. In fact, only one study on GABGRP (glass-aluminum-banana-glass-reinforced plastic) [27], made of GFRP, banana fibers and some sort of relatively thick aluminum lattice, can be found. This material exhibited better strength and impact resistance than a benchmark GFRP; however, its electrical conductivity was not investigated.

### 2.1. Constituent Properties and Compatibility

**Figure 2:**
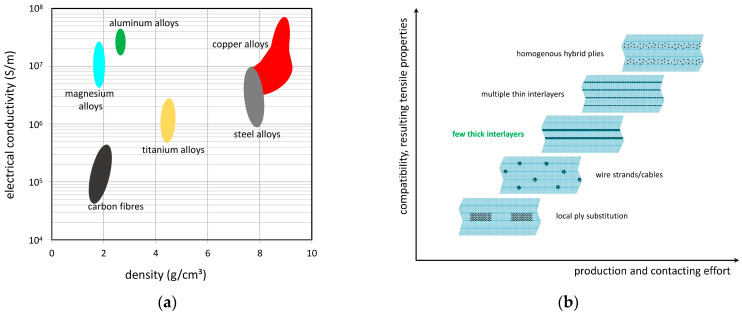
(**a**) Electrical conductivities and densities of carbon and metal fiber candidates [28,29]; (**b**) different approaches to metal fiber integration.

The use of thin aluminum fibers to add electrical conductivity to a GFRP material is interesting for several reasons. While conductive SFRPs lose their lightweight design potential with increasing steel content, this is less the case when using aluminum fibers due to their much lower density ($2.7\;g/{\mathrm{cm}}^{3}$ instead of $7.8\;g/{\mathrm{cm}}^{3}$). Furthermore, with an electrical conductivity ($3.83\times 10^{7}\;S/m$) of about 65% that of copper ($5.96\times 10^{7}\;S/m$), aluminum is a much better conductor than most ferrous metals, such as heat-treatable steel ($1.45\times 10^{6}\;S/m$). A correlation of the electrical conductivities with the mass densities is depicted in Figure 2a. As indicated by the plot, aluminum and magnesium both provide low densities and high conductivities.

Magnesium, however, is an inferior candidate due to flammability issues associated with thin magnesium products, its lower mechanical properties, and the lack of fiber or fine-wire-wrought materials. An overview of representative mechanical properties of aluminum, E-glass and epoxy materials is summarized in Table 1. All values are reference values representing the material class. Concerning the elastic moduli, the E-glass and aluminum have quite similar properties. Their failure behavior and strength, however, differ substantially. In contrast to GFRP, which practically exhibits a linear elastic stress–strain behavior until rupture (loaded parallel to the fibers), aluminum shows a pronounced elastic-plastic behavior above its elastic limit. This, of course, depends on the aluminum alloy and the heat treatment. As the maximum linear-elastic strains of aluminum (max  0.7%) are much smaller than those of E-glass (3–4%), the stress–strain compatibility of both materials is limited. In fact, deforming a hybrid composite material above the elastic limit of the aluminum is assumed to cause irreversible plastic deformation of the aluminum fibers and permanent internal stresses after unloading. Repeated loading and unloading may initiate interfacial debonding of the aluminum fibers due to interfacial shear stresses. Consequently, adding oriented quasi-endless aluminum fibers is assumed to diminish the allowable strain range. However, the impact on the overall composite behavior and resulting properties is difficult to predict and is therefore investigated experimentally.

Since the longitudinal coefficient of thermal extension of aluminum ($25\;\times 10^{-6}\;1/K$) is about five times higher than that of E-glass fibers ($5\;\times 10^{-6}\;1/K$), temperature-induced internal stresses are likely to arise. This applies to both hot curing during manufacturing, as well as later temperature exposure during service. In contrast to the hybridization of carbon-fiber-reinforced plastics, electrochemical compatibility is not an issue when using glass fibers.

In addition to mechanical compatibility, both the geometric properties (diameters) of the aluminum fibers and the fiber distribution can affect the resulting properties of the composite with a given aluminum content. Concerning the fiber diameter, the integration of aluminum fibers with diameters similar to those of the glass fibers is assumed to be advantageous with respect to local homogeneity and the resulting strain field on the micro scale. This also holds true for fiber distribution on the scale of the single ply. Figure 2b illustrates five different methods of wire conductor integration, ranging from local ply-substitution (locally clustered aluminum fibers), over-distributed strands and thick and thin aluminum fiber interlayers to homogeneous hybrid plies. With increasing homogeneity, the geometrical compatibility and resulting composite strength are assumed to increase. Unfortunately, increased efforts related to manufacturing and electrical contacting are expected as well. Thus, within this work, an interlayer approach using multiple unidirectional aluminum fiber interlayers is investigated. This type of integration allows for a greater homogeneous fiber distribution than, for example, strand-based approaches. Furthermore, the aluminum fiber interlayers, with a thickness of about one wire diameter, can be contacted more conveniently than, for example, hybrid single plies with both aluminum and non-conducting glass fibers.

### 2.2. Properties of the Unidirectional AlFGFRP

Fundamental properties such as the elastic modulus, mass density or electrical conductivity of unidirectional composites can be estimated analytically by means of the rule of mixture approaches [29,32,33]. Homogenized properties of the idealized composite are modelled assuming either a parallel connection of the constituents (fiber-parallel direction) or a series connection (transverse direction), and by taking into account their individual properties ($X_{i}$) and volume fractions ($\varphi_{i}$, $\sum_{i}\varphi_{i}=1$). Equation (1) holds true for the fiber-parallel elastic modulus ($E_{11}$), the electrical resistivity ($r_{11}$) and, not depending on the fiber-orientation, the density (ρ):

(1) $$X=\sum_{i}X_{i}\cdot \varphi_{i}\;$$

Concerning the fiber transverse direction, only the modulus is of relevance, as the transverse electrical conductivity is dominated by the insulating epoxy matrix and glass fibers, with the aluminum fibers not interconnected under idealized conditions. The effective transverse modulus, *E*_22_, of the three-phase composite can be approximated by Equation (2):

(2) $$E_{22}={\left(\sum_{i}\frac{\varphi_{i}}{E_{2,i}}\right)}^{-1}$$

To account for the lateral contraction of the matrix restrained by the much stiffer fibers, an increased matrix modulus ${\bar{E}}_{m}=E_{m}{\left(1-\nu_{m}^{2}\right)}^{-1}$ is used.

**Figure 3:**
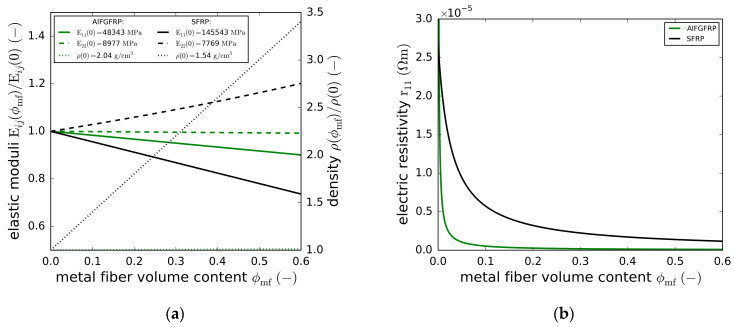
Properties of the AlFGFRP and SFRP for different metal volume fractions: (**a**) normalized elastic moduli and density; (**b**) electrical resistivity.

Figure 3a,b depicts the predicted elastic moduli, density and electrical resistivity of the unidirectional AlFGFRP and SFRP configurations. For a technically relevant total fiber volume fraction of $\phi_{f}=0.6$, the metal fiber fraction varied from $\phi_{\mathrm{mf}}=0.0$ (reference FRP) to $\phi_{\mathrm{mf}}=0.6$ (only metal fibers). All results were normalized to the properties of the reference FRP. The input constituent properties are given in Table 2. Comparing the impact of the metal fiber integration on both material systems (AlFGFRP and SFRP) revealed significant differences. Concerning the fiber-parallel stiffness, replacing the glass fibers with aluminum fibers caused only minor stiffness decreases (about 10% for $\phi_{f}=\phi_{\mathrm{mf}}=0.6$) due to the similar elastic properties of the constituents. Substituting the carbon fibers with steel fibers, in contrast, caused significant stiffness decreases (about 25% for $\phi_{f}=\phi_{\mathrm{mf}}=0.6$). The situation was different for the transverse direction. While steel integration resulted in a stiffening due to the low transverse modulus of the carbon fibers, aluminum integration did not cause noticeable changes in $E_{22}$. Concerning the density, the impacts on the composite density were negligible when adding aluminum to the GFRP. Adding steel, in contrast, caused the density to increase by a factor close to 3.5 for $\phi_{f}=\phi_{\mathrm{mf}}=0.6$.

Apart from the undesired density increases and stiffness reductions, metal integration may reduce the composite strengths. Accordingly, the metal content should be as low as possible, but as high as necessary, with respect to the electrical properties. For an SFRP, Hannemann et al. [26] identified steel fiber volume contents between 10% and 20% as a reasonable compromise. However, the density almost doubles from CFRP to SFRP containing 20% steel. In the case of the AlFGFRP (Figure 3b), volume fractions ranging between 5% and 10% seem to be reasonable. In this study, an aluminum volume fraction of 10% was chosen.

## 3. Materials and Methods

In the following section, the laminate and specimen fabrication methods are described, along with a summary of the important information on the used wrought materials and composite constituents. Concerning the mechanical test program, a brief overview of the standard test methods and a more detailed depiction of the combined load setup (electrical and mechanical load) are provided. Additionally, the measurement techniques used for the condition/damage monitoring are outlined.

### 3.1. Aluminum-Fiber-Reinforced GFRP (AlFGFRP)

Based on the material concept depicted in Section 2, a hybrid polymer composite containing E-glass fibers and aluminum fibers was fabricated by means of a specifically designed fabrication method. Aluminum fibers were integrated as pre-impregnated interlayers, as this is a convenient way of handling large amounts of fine wire and of realizing a rather homogeneous distribution that is not strand-based. In addition, this also has advantages for the electrical contacting.

#### 3.1.1. Fabrication Method

**Figure 4:**
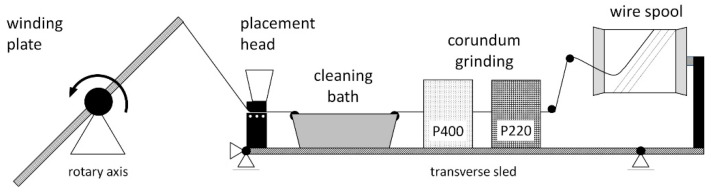
Fine wire-winding machine.

A fine wire-winding machine was specifically designed and set up for the fabrication of the unidirectional aluminum single plies. Due to the low rupture load of the wire (between 0.5 N and 2.0 N  depending on the diameter, alloy and state of annealing), automated processing is challenging, including unspooling, wire surface preparation and cleaning, placement and winding. A schematic of the wire processing is depicted in Figure 4. After the wire is pulled from the spool, its surface is mechanically abraded using a two-step corundum bath. In the next step, the wire is cleaned using alcohol. Residual alcohol on the wire is stripped off mechanically and actively vaporized. The placement of the wire on the winding plate (length 350 m, width 220 mm) is realized by means of a placement head. The maximum winding speed is 120 rpm (average pulling speed of 1.4 m/s), with a transverse axis speed of 0.1 mm per revolution.

**Figure 5:**
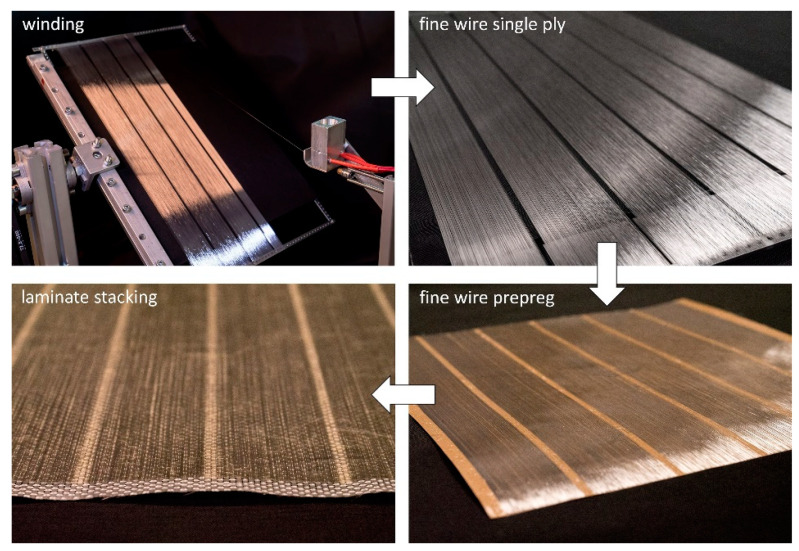
Steps of the aluminum fiber integration.

The main steps of the AlFGFRP fabrication method are depicted in Figure 5. The first step is fine-wire winding. The resulting single plies can either be continuous or segmented (as shown in Figure 5), forming discrete electric tracks. After the winding process, a resin film is molded onto the aluminum single layer. As both sides of the winding plate are used, two prepreg sheets can be produced at one time. The prepreg sheets are then stacked with dry fabrics and, if needed, an additional resin film. The final layup is then impregnated and cured by means of autoclave-based resin film infusion (180 °C, 3 h, 300 kPa pressure).

#### 3.1.2. Composite Constituents and Configurations

Two different laminate layups, a GFRP reference laminate and an AlFGFRP layup made from glass fabric (G), resin film (R) and combined aluminum-fiber resin film (AR), were investigated. The stacking sequences were [G/R/G]_4_ for the reference laminate and [G/AR/G]_4_ for the AlFGFRP. In both layups, all plies were oriented under 0° with the warp threads and the aluminum fibers parallel to the *x*-axis of the laminate. The y-direction denotes the direction of the transverse weft threads. Regarding the AlFGFRP layup, different aluminum wires and spacings were used, depending on the experimental objective.

**Figure 6:**
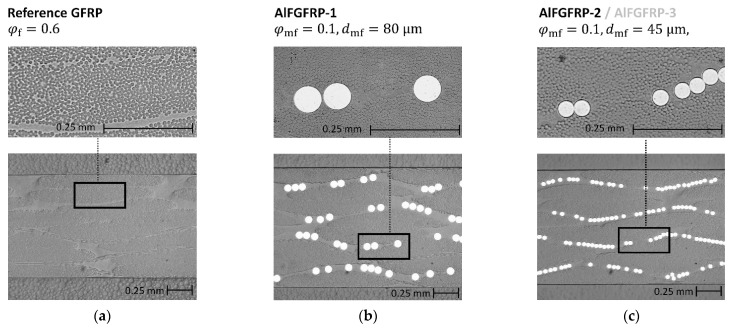
Overview of the three manufactured laminated configurations: (**a**) reference GFRP; (**b**) AlGFRP-1, containing work-hardened wire; (**c**) AlFGFRP-2 (work-hardened) and, equivalently, AlFGFRP-3 (soft-annealed).

Concerning the constituents, an unreinforced epoxy resin film, MTM44-1 ($200\;g/m^{2}$, Solvay, Brussels, Belgium), was used for impregnating the unidirectional (warp-reinforced) E-glass plain weave fabrics ($220\;g/m^{2}$, chain EC9-68 × 5, weft EC7-22, finish GI6224). For the AlFGFRP materials, three different types of wires were used. A work-hardened (H18) 5019 super fine wire with a diameter of 45 µm (J.G. Dahmen GmbH & Co., KG, Iserlohn, Germany) was used for the main static and fatigue experiments. This wire has a rather linear stress–strain relationship, with rupture occurring right after the elastic limit is passed. Additionally, a soft-annealed variant (with a much lower yield limit) was used for investigating the effects of plasticity. In the pre-tests, the stress–strain behavior of both wires was characterized by applying the single-fiber tension cardboard method (ASTM D 3379–75 [34]). As the wires inevitably undergo heat treatment (and potential softening) during autoclave processing, the properties of the delivery states are not representative. Thus, a replacement heat treatment (180 °C, 3 h) was conducted prior to the single wire testing. The single wire strengths and strains are summarized in Table 3. Furthermore, a soft-annealed aluminum alloy 5019 fine wire (Gutmann Aluminium Draht GmbH, Weißenburg in Bayern, Germany) with a diameter of 80 µm was used to assess the effects of the wire diameter and spacing by means of transverse tensile tests. Sample cross-section micrographs of all three laminate configurations are depicted in Figure 6.

#### 3.1.3. Test Specimens

**Figure 7:**
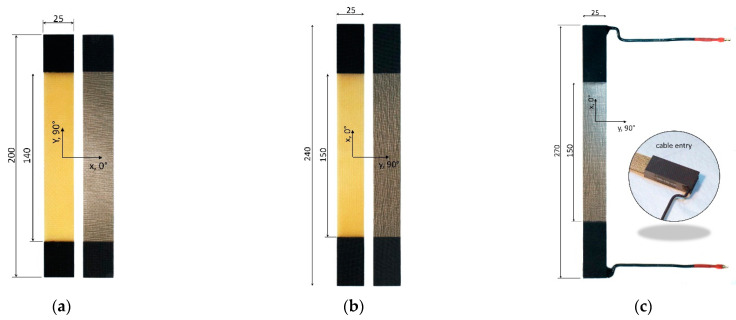
Overview of the test specimens (all dimensions in mm): (**a**) transverse tension; (**b**) longitudinal tension, (**c**) longitudinal tension with electrical contacts.

Three different types of specimens, as depicted in Figure 7, were fabricated. Concerning the characterization of both the reference laminate and the AlFGFRP laminates, transverse (90°) tension specimens (Figure 7a) and longitudinal (0°) tension specimens (Figure 7b) were fabricated according to DIN EN ISO 527-5 [35]. However, due to the limiting dimensions of the winding plate, slight changes to the tab and gauge lengths had to be made. For the combined electrical-mechanical tests, a specimen containing electrical contacts was designed (Figure 7c). While the gauge length was equal to the standard length, end tabs were longer and much thicker (6 mm tab laminate thickness) due to the electrical contacts. The connection wire was a solid copper wire, AWG 12 ($3.31\;{\mathrm{mm}}^{2}$), with a current rating of 20 A. Soldered 4 mm banana gold connectors were used for connecting the specimens to the power electronics. Wire entries were sealed with high-temperature-resistant silicone for the insulation and to keep the wire in place in the case of soldering point failure.

### 3.2. Electrical Resistance Measurement

The electrical resistances were determined using a Potentiostat/Galvanostat Model 263A (four-probe method, Princeton Applied Research, Oak Ridge, TN, USA). Potentiodynamic scans were performed in the case of the single wire and the mechanically unloaded multifunctional material. For the single wire, the voltage was swept between −0.01 V and 0.01 V (1 mV/s) with respect to the open circuit. For the multifunctional material, the voltage range was from −0.25 V to 0.25 V at a rate of 10 mV/s. The mean slope of the V-I graph gives the electrical resistance. During the mechanical tests of the multifunctional material, potentiostatic measurements were conducted at a pre-set potential of 0.25 V and with a sampling rate of 200 1/s. The electrical resistance was calculated using Ohm’s Law.

### 3.3. Thermography

A high-performance thermal camera (Model X8503sc, FLIR, Wilsonville, OR, USA) with a sensor resolution of 1280 × 1024 pixels^2^ was used during the mechanical, electrical and combined tests. Concerning the mechanical fatigue tests, the camera was used primarily for damage monitoring and to reveal differences in the heating and damage behavior of the GFRP reference laminate and the AlFGFRP materials. Furthermore, the current-induced heating of the mechanically unloaded AlFGFRP was investigated. During the combined electrical-mechanical tests, the temperature was monitored to reveal heating effects related to the damage of the electrical conductors and final specimen failure. A 10 °C to 90 °C calibration interval was used. During the fatigue tests, single images were captured to reduce the amount of data. The capturing rate was 10/s , 1/s  and 0.01/s  depending on the test duration.

### 3.4. Static and Fatigue Testing

**Figure 8:**
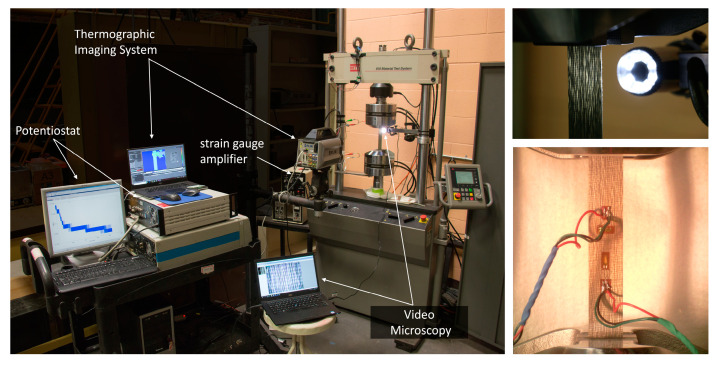
Overview of the test setup for the static tensile tests and cyclic tension–tension fatigue tests, including resistance measurements (potentiometry), thermographic imaging, online video microscopy and strain gauge measurements.

The static and fatigue testing was conducted using a servo-hydraulic testing machine (Model 810, MTS, Eden Prairie, MN, USA) with a load capacity of 100 kN. Depending on the laminate configuration, different measurement and damage monitoring techniques were applied. For the static material characterization, longitudinal and transverse tensile tests were conducted according to DIN EN ISO 527-5 [35] for both the reference GFRP and the AlFGFRP configurations. During these tests, strain gauge measurements and video microscopy were conducted. In the case of the electric conductive material, additional potentiostatic measurements were conducted to determine the resistance. During the tension–tension fatigue tests (R=0.1), the self-heating of the specimens was investigated by means of thermography. An overview of the test set-up showing the measurement techniques is depicted in Figure 8. The tests were conducted with a load frequency of 1 Hz,  except for the lowest load level (2 Hz).

For the damage monitoring, the dynamic modulus $E_{\mathrm{dyn},x}$ was calculated from the cyclic force and strain maxima and minima (tip–tip modulus, Equation (3)). Furthermore, the hysteretic loss energy (Equation (4)) was calculated for each load cycle, represented by the surface enclosed by the loading and unloading curves in the stress–strain diagram. The relative loss energy ($U_{\mathrm{loss}}/U_{\mathrm{loading}}$) was used as the comparative measure for the energy dissipation due to the composite damage and non-elastic material deformation of the metallic phase.

(3) $$E_{\mathrm{dyn},x}=\frac{\sigma_{\max}-\sigma_{\min}}{\varepsilon_{\max}-\varepsilon_{\min}}$$

(4) $$U_{\mathrm{loss}}=U_{\mathrm{loading}}-U_{\mathrm{unloading}}$$

For the statistical evaluation of the of obtained SN data, a linear relationship between the logarithm of the stress (*S*) and cycles to failure (*N*) was used (Equation (5)). In addition, 95%  prediction limits $N_{P\%}^{\pm }$ (Equation (6)) were determined, according to Schneider and Maddox [36]:

(5) log N=logA−m logS

(6) $$\log N_{P\%}^{\pm }=\left(\log A+m\log S\right)\pm t\hat{\sigma }\sqrt{1+\frac{1}{n}+\frac{{\left(\log S-\bar{\log S}\right)}^{2}}{\sum_{i=1}^{n}{\left(\log S_{i}-\bar{\log S}\right)}^{2}}}$$

### 3.5. Combined Electrical and Mechanical Testing

**Figure 9:**
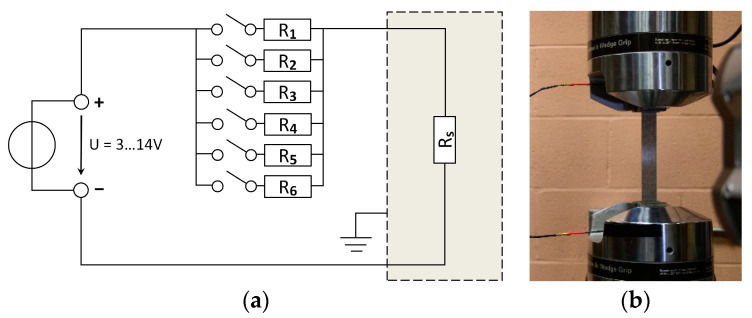
(**a**) Electrical circuit diagram used for applying the electrical loads, where R_1_ to R_6_ are power resistors and R_S_ represents the specimen; (**b**) specimen installation with electrical wiring.

The aim of this unique test series was to investigate the impact of the electric current on the mechanical (quasi-static and fatigue) behavior of the AlFGFRP material. Therefore, the contacted specimen type was used to complete the electric circuit, depicted in Figure 9a. The circuit consisted of a DC power supply (3 V–14 V, 40 A, Model 1692, B+K Precision, Yorba Linda, CA, USA) and a parallel connection of six switchable power resistors (4 Ω each). This setup was created to study the current-carrying capability of the material. The voltage, as well as the total resistance, were adjusted to obtain the desired current passing through the specimen. Figure 9b shows a clamped specimen with the electrical wiring.

As temperature is known to affect the fatigue life of FRP materials [37,38], the control of the specimen’s heating is an important matter. In the case of the AlFGFRP material, with its mismatch between the thermal expansion coefficients of the constituents, the temperature effects were assumed to be even more critical. On the one hand, to assess the potential detrimental effects of the electric current, ohmic heating should be avoided or, at least, kept low by limiting the current. On the other hand, the current passing through the material must be relevant and representative of technical applications. Although the heating of conventionally isolated cables is tolerable up to 70 °C  [39], this temperature is considered too high to avoid temperature effects in AlFGFRP laminates. Instead, a much lower temperature limit of 40 °C  was defined. The specimen surface temperature was monitored by means of thermography.

### 3.6. Simplistic Electrical Resistance Model

**Figure 10:**
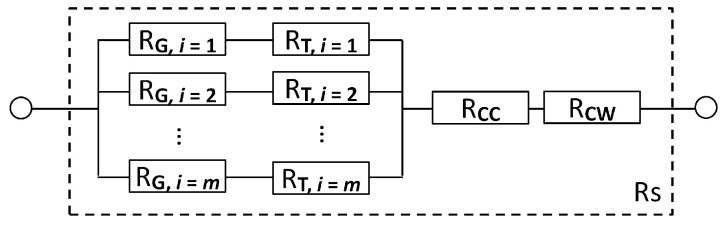
Idealized circuit diagram for the approximation of the electrical resistance of a contacted specimen, consisting of aluminum fibers in the gauge length ($R_{G}$) and the end tabs ($R_{T}$), and additional resistances for the crimping connections ($R_{\mathrm{CC}}$) and the copper wire ($R_{\mathrm{CW}}$).

Assuming several idealizations concerning the laminate architecture and physical properties, a simplistic model is proposed for estimating the effective electrical resistance, resistance degradation and ohmic heating. Concerning the architecture of the AlFGFRP laminate, it is assumed that the aluminum fibers are straight, parallelly aligned and not interconnected in the transverse direction. The total electrical resistance of a laminate unit cell can thus be idealized as the parallel connection of a discrete number of equal ohmic resistors. Each ohmic resistor $R_{G,i}$ represents a single conductor of a given diameter $d_{R}$, length $l_{R}$ and resistivity $r_{R}$,. In the case of the contacted specimen type (Figure 7c), two different regions (G: gauge region, and T: tab region) must be differentiated, as illustrated in Figure 10. While the total electrical resistance is assumed to be temperature- and strain-dependent in the gauge section ($R_{G}$(ϑ,ε)), only the influence of the temperature must be considered in the tabbed region ($R_{T}\left(\vartheta \right)$). Furthermore, two additional resistors representing the two crimping connections ($R_{\mathrm{CC}}$ = const.) and the two copper connection wires ($R_{\mathrm{CW}}$(*T*)) are considered. Wire ruptures in the specimen gauge length due to mechanical loading are accounted for by reducing the number of parallel resistors (m).

The total resistance, according to the equivalent resistance model, can thus be calculated by means of Equation (7). The model yields reasonable results as long as all conductors are intact. In the case of damage, however, the analytical model overestimates the decreases in the resistance, since conductor interruptions are, to some extent, compensated by the existence of transverse interconnections.

(7) $$R_{S}\left(\vartheta ,\varepsilon \right)=\frac{R_{G}\left(\vartheta ,\varepsilon \right)+R_{T}\left(\vartheta \right)}{n}+R_{\mathrm{CC}}\left(\vartheta \right)+R_{\mathrm{CW}}\left(\vartheta \right)$$

The resistance change of a single mechanically stressed cylindrical fiber is approximated by incorporating the longitudinal wire elongation and a change in the diameter due to the Poisson effect (ν=0.35 for aluminum). The resistance of the elongated conductor is:

(8) $$R\left(\varepsilon \right)=\rho \frac{l_{0}\left(1+\varepsilon \right)}{\pi {\left(\frac{d_{0}}{2}\left(1-\nu \varepsilon \right)\right)}^{2}}$$

Concerning the temperature dependency, the ohmic resistance at a discrete temperature [ϑ] = °C is approximated by means of the following linear relationship and the temperature coefficient β, $\left[\beta \right]=1/K$ [40]:

(9) $$R\left(\vartheta \right)=R_{0}\left(1+\beta \left(\vartheta -\vartheta_{0}\right)\right),\;\mathrm{with}\;\beta ={3.77\times 10}^{-3}$$

The equilibrium temperature due to the ohmic heating of the gauge section is approximated by means of Equation (10) [41]. As the voltage drop over the gauge section could not be measured in the experiments, the power is calculated using the measured current and a theoretical gauge resistance $R_{G}/m$. $A_{G}$ denotes the surface area of the gauge section and α is the heat transfer coefficient for the passive convection ($\left[\alpha \right]=W/(m^{2}K)$):

(10) $$∆\vartheta =\frac{I_{\mathrm{const}}{}^{2}\cdot R}{\alpha A_{G}}\;\mathrm{with}\;5\;\leq \alpha \leq 15$$

## 4. Results

### 4.1. Tensile Properties

Quasi-static tensile tests were conducted for all four laminate configurations. Thereby, the reference laminate GFRP and the AlFGFRP-2 configuration (work-hardened) were tested under longitudinal and transverse tension. AlFGFRP-3 (soft-annealed) was only tested in the fiber-parallel direction, as the annealing was assumed to primarily affect the longitudinal laminate properties. In contrast, the diameter of the aluminum fibers was assumed to be important for the transverse properties. Therefore, AlFGFRP-1 (work-hardened, 80 µm) and AlFGFRP-2 (work-hardened, 45 µm), both containing the same aluminum volume fraction, were tested under transverse tension to reveal the impacts of the metal fiber diameter. Three specimens were tested for each laminate configuration and load direction.

**Figure 11:**
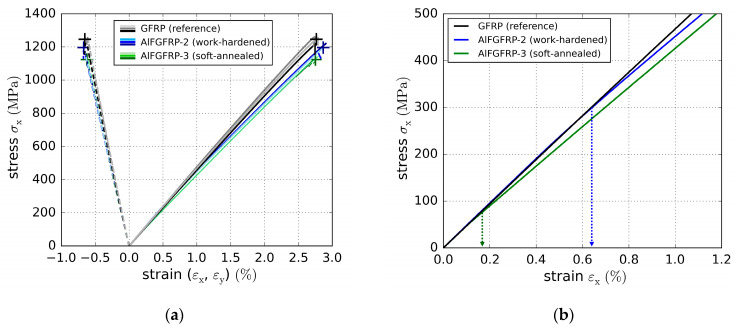
Stress–strain results of the reference laminate and aluminum-reinforced laminates containing the soft-annealed and work-hardened wire tested under fiber-parallel tension: (**a**) original stress–strain plot showing the longitudinal (solid lines) and transverse stress–strain curves (dashed lines); (**b**) enlarged plot of the lower stress–strain region.

Figure 11a depicts the stress–strain curves for the longitudinal elongation and lateral contraction. All specimens of a configuration are marked by similar colors. The cross-shaped markers indicate the average stress and strain at rupture of a configuration. For the fiber-parallel direction, the stress–strain curves of the GFRP and the two configurations with the thin metal fibers (45 µm) are quite linear across the full range of the strain until final failure (subscript: f). The GFRP reference laminate fails at an average longitudinal stress of $\sigma_{x,f}^{GFRP}=1246\;\mathrm{MPa}$ ($\varepsilon_{x,f}^{GFRP}=2.76\%)$. AlFGFRP-2 (work-hardened) shows a 4% lower average failure stress of $\sigma_{x,f}^{\mathrm{AlFGFRP}\text{-}2}=1197\;\mathrm{MPa}$. The failure strain ($\varepsilon_{x,f}^{\mathrm{AlFGFRP}\text{-}2}=2.85\%$) is higher by a factor of 1.03. In the case of the configuration containing the soft-annealed aluminum fibers (AlFGFRP-3), the failure stress is reduced by about 10% ($\sigma_{x,f}^{\mathrm{AlFGFRP}\text{-}3}=1124\;\mathrm{MPa}$) compared to the reference laminate. In both cases, the strength reduction is due to the aluminum fibers having lower tensile strengths (Table 3) than the glass fibers (cf. representative values, given in Table 1). The failure strain does not change ($\varepsilon_{x,f}^{\mathrm{AlFGFRP}}=2.76\%)$. Standard deviations are lower than 2% for all longitudinal stress/strain results. The elastic modulus of the configuration containing the work-hardened wire (AlFGFRP-2) ($E_{x}^{\mathrm{AlFGFRP}\text{-}2}=\text{47,735}\;\mathrm{MPa}$) is slightly higher than the modulus of the GFRP reference ($E_{x}^{\mathrm{GFRP}}=\text{47,211}\;\mathrm{MPa}$). Notably, this is not in line with the analytical estimations (Figure 3), predicting a slight stiffness decrease of less than 2% for a metal volume content of 10%. However, as the standard deviations are about 1.5%, and as the analytical calculation does not take into account the transverse warp fibers, geometrical imperfections and manufacturing effects, these results are considered reasonable. In contrast, in the case of the soft-annealed wire (AlFGFRP-3), a 10% modulus reduction was found ($E_{x}^{\mathrm{AlFRC}\text{-}3}=\text{43,279}\;\mathrm{MPa})$. The averaged stress–strain curves are depicted in Figure 11b for strains of up to 1.2%. In this graph, differences in the stress–strain behavior can be observed. In the case of the work-hardened aluminum fiber, the stress–strain curves are non-linear and diverge from the reference curve at strains above approximately 0.65%. For the soft-annealed wire, curves start to diverge much earlier, at strains above 0.17%. This is assumed to result from the elastoplastic deformation of the aluminum fibers when exceeding the elastic limit. The elastic limits (0.62%  and 0.19%) identified by the single wire tension pre-tests (Table 3) support this explanation. Higher non-linearity caused by elasto-plastic deformation may also be an explanation for the low elastic-modulus, which is based on the 0.05% to 0.25% strain range. A summary of the most important quantitative results is given in Table 4.

**Figure 12:**
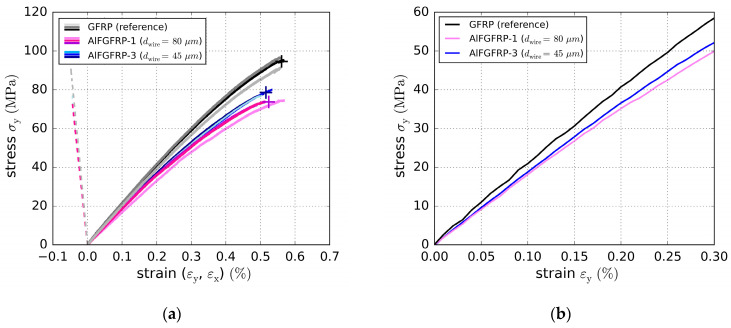
Stress–strain results of the reference laminate and aluminum-reinforced laminates containing work-hardened wires of different diameters tested under fiber-transverse tension: (**a**) original stress–strain plot showing the longitudinal and transverse stress–strain curves; (**b**) enlarged plot of the lower stress–strain region.

The stress–strain curves of the transverse tensile tests are depicted in Figure 12a,b. Due to the low fraction of transverse glass fibers, the transverse deformation behavior is much more matrix-dependent and, thus, less linear-elastic. These results indicate that the diameter of the metal fiber is important for the transverse laminate properties. It was assumed that a thin metal fiber would not affect the strength as much as a thick one due to its better geometrical compatibility. In fact, the AlFGFRP-3 containing the 45 µm aluminum fiber had 83% the transverse tensile strength of the GFRP reference laminate, whereas for the thicker wire (AlFGFRP-1, 80 µm), the transverse strength was lower (78% of the reference strength). However, in both cases, the strength was significantly degraded, presumably due to weak matrix–aluminum fiber interface bonding. Strains at rupture and elastic moduli were less affected. The results are summarized in Table 5.

### 4.2. Fatigue Behavior

In this section, the experimental results of the fatigue tests (tension-tension, R=0.1) are presented. Fatigue tests were conducted for the longitudinal (fiber-parallel) direction only, focusing on the potential impacts of the elasto-plastic deformation behavior of the aluminum. The tests were conducted at four different load levels with cyclic maximum loads of 80% (997 MPa), 60% (748 MPa), 50% (623 MPa) and 30% (374 MPa) of the quasi-static longitudinal strength of the reference laminate (1264 MPa). Due to the limited number of available specimens, only one to two specimens per load level could be tested.

**Figure 13:**
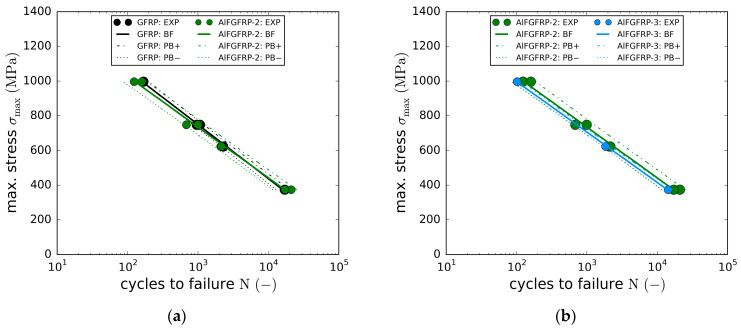
SN curves with regression and prediction bands: (**a**) comparison of the AlFGFRP containing work-hardened aluminum (AlFGFRP-2) with the GFRP reference laminate; (**b**) SN curve comparison revealing the impact of the wire condition, work-hardened (AlFGFRP-2) and soft-annealed (AlFGFRP-3).

Figure 13a depicts the SN curves (linear-log) of the GFRP reference (black) and the AlFGFRP-2 (work-hardened) laminates. Both curves are very close to each other, with their 95% prediction bands nearly matching. Due to the small number of specimens tested, the load-level-specific differences in fatigue life cannot be discussed. However, concerning all the specimens, an average fatigue life reduction of 6.5% was found for the AlFGFRP-2, which is in line with the results of the quasi-static tests. Overall, the integration of the work-hardened wire did not cause significant fatigue life reductions. In the case of the soft-annealed wire, the fatigue life reductions were found to be more distinct, as depicted in Figure 13b. On average, the fatigue life changed by about −18.6% compared to the material containing the work-hardened wire and −21% compared to the reference GFRP. Higher fatigue life reductions were assumed to be caused by the increased plastic deformation and residual elongation of the aluminum wires, resulting in internal stresses and the pronounced initiation of local micro-scale interfacial debonding, e.g., in the vicinity of aluminum fiber ruptures. It is worth pointing out that even the reference material had a surprisingly poor fatigue performance. Even at the lowest load amplitude of 30% of the static tensile strength, the fatigue life was limited to about 20,000 cycles. This is rather untypical for quasi-unidirectional E-glass/epoxy GFRP materials and much below the expected values. Most GFRP materials of this kind reach 1 million or more cycles when tested at similar load levels. The analysis of the damage behavior (Section 4.3) indicated that the autoclave processing and the resulting high compaction may have caused this poor fatigue performance.

**Figure 14:**
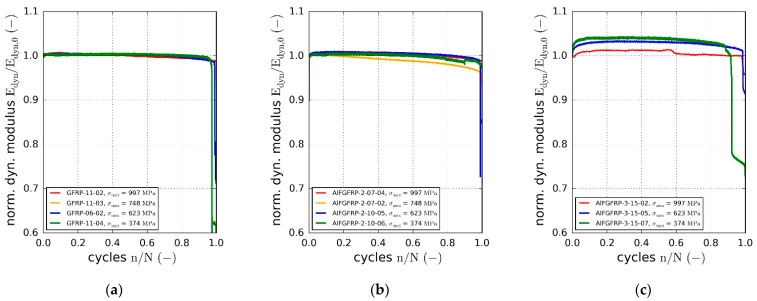
(**a**) Normalized dynamic modulus (tip–tip) against the normalized fatigue life: (**a**) reference laminate; (**b**) laminate with work-hardened aluminum fibers; (**c**) laminate with soft-annealed aluminum fibers.

Based on the cyclic force and displacement data, the dynamic modulus was calculated using Equation (3). The normalized dynamic modulus is plotted against the normalized cycle number for all three material configurations in Figure 14a–c. Due to the mainly unidirectional fiber orientation (0°), no significant degradation of stiffness occurred until the sudden final failure. This is typical for fiber-dominated material orientations. However, even though the stiffness of the glass fibers does not change under cyclic loading, a slight stiffness degradation resulted from setting effects, matrix micro-damages and the change in the transverse contraction behavior. Overall, the degradation behavior of the AlFGFRP-2 did not exhibit significant differences. However, a slight increase in the stiffness during the first 10% of the fatigue life was observed. During the second half of the fatigue life, the stiffness degradation seemed to be slightly more pronounced in all the specimens. The observed initial stiffening effect followed by the stiffness degradation became more apparent in the case of the third configuration containing the soft-annealed wire. Stiffness increases about 4% during the first 10% to 20% of the fatigue life, in the case of the lowest load level. It seems that the stiffening effect increased with decreasing load level. With damage related degradation lower than or equal to the initial stiffening, the final rupture was still observed to occur at elevated stiffness levels. As the stiffening effect was more distinct in the case of the soft-annealed wire, this behavior was assumed to be caused by the plastic deformation of the aluminum under cyclic loading.

**Figure 15:**
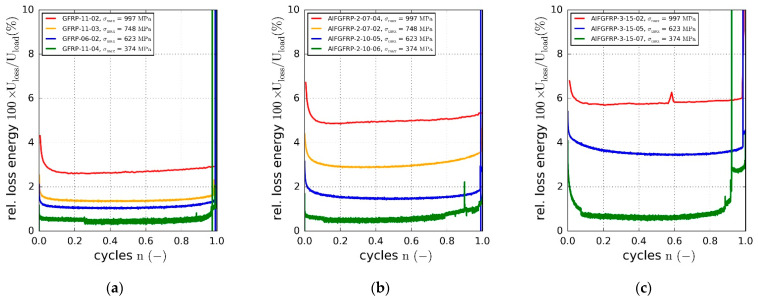
Relative loss energy against the normalized fatigue life: (**a**) reference laminate; (**b**) laminate with work-hardened aluminum fibers; (**c**) laminate with soft-annealed aluminum fibers.

**Figure 16:**
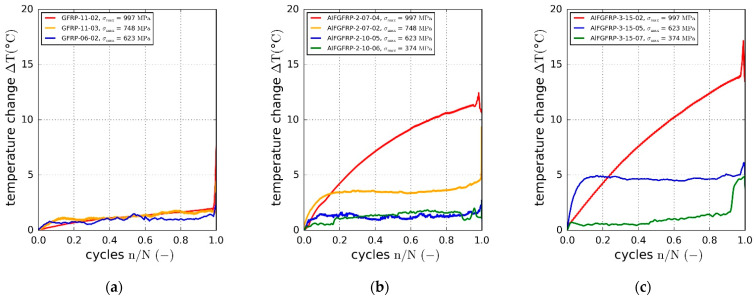
Surface temperature change against the normalized fatigue life: (**a**) reference laminate; (**b**) laminate with work-hardened aluminum fibers; (**c**) laminate with soft-annealed aluminum fibers.

Hysteretic loss is another indicator of irreversible deformation processes due to plasticity or fatigue damage of the composite. The relative loss energy is plotted against the normalized cycle number in Figure 15a–c. The relative dissipated energy increased with the cyclic load amplitude. Compared to the reference material, the relative loss energy was found to increase due to the metal fiber integration. While about 3% of the introduced strain energy was dissipated at the highest load level in the case of the reference GFRP, the relative loss increased to about 5% for AlFGFRP-2 and 6% for AlFGFRP-3. This tendency can also be seen in the heating curves, showing a change in the specimen surface temperature with increasing cycle number (Figure 16a–c). It should be noted that the heating behavior of the reference specimen tested at the highest load level did not correspond with the plotted loss energy. A reason for this effect could not be ascertained. Nevertheless, overall, the heating curves are in accord with the loss energy curves.

All three data sets (dynamic modulus, relative loss energy and temperature change) indicate the presence of plastic deformation, damage of the aluminum fibers and, potentially, induced/related micro-scale damages of the matrix or fiber–matrix interfaces.

### 4.3. Damage Mechanisms

To gain an understanding of the damage mechanisms and their development under both types of loading, microscopy was conducted in situ (during testing), as well as post-failure. Since the damage mechanisms of the pure GFRP laminate were hardly visible, due to its optical properties, only the AlFGFRP material was examined in detail by microscopy.

**Figure 17:**
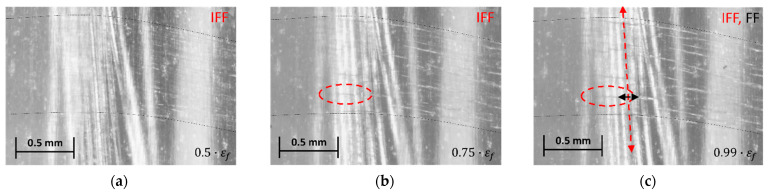
Representative micrograph sequence (reflected light in situ microscopy of the specimen surface), illustrating the occurrence of transverse inter-fiber cracking (IFF) and fiber-fracture (FF) in the AlFGFRP material under static loading. The images refer to 50% (**a**), 75% (**b**) and 99% (**c**) of the strain to rupture.

Under static loads, the AlFGFRP laminates showed initiation and accumulation of matrix cracks (inter-fiber cracks, IFF) only in the transversely oriented weft tows. The micrographs in Figure 17a–c depict reflected light in situ micrographs at relative strains of approximately 50%, 75% and 99% of the strain to rupture, respectively. The damage accumulation at the crossings of the warp and weft was observed to trigger the final failure. Originating from these crossing points, longitudinal splitting and subsequent fiber failure resulted in the specimens’ overall disintegration due to abrupt energy release. In situ microscopy did not provide evidence of aluminum fiber breakage before the final specimen rupture. Encircled transverse damages (red) were identified as inter-fiber cracks accumulating in the weft tow.

**Figure 18:**
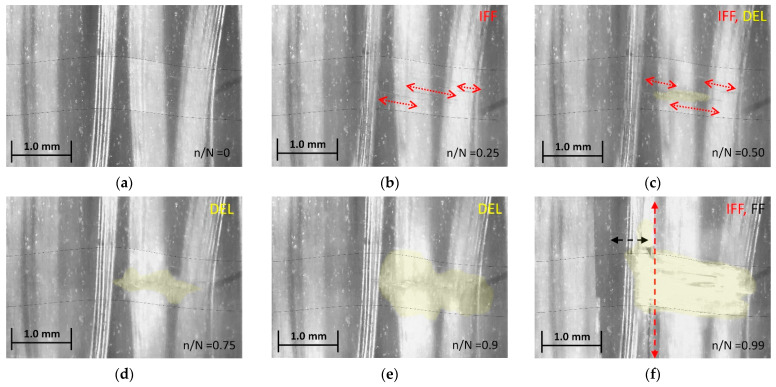
Representative micrograph sequence (reflected light in situ microscopy of the specimen surface), illustrating the occurrence of transverse inter-fiber cracking (IFF), delamination (DEL) and fiber-fracture (FF) in the AlFGFRP material under fatigue loading. The images depict the damage states after 0% (**a**), 25% (**b**), 50% (**c**), 75% (**d**), 90% (**e**) and 99% (**f**) of the cycles to failure.

In the cyclic tests, inter-fiber cracking in the weft tow (Figure 18a–c) was identified to be the first and most widespread damage mechanism. However, before the occurrence of fiber ruptures and final failure, local delamination of the weft tows (Figure 18c–f) was observed. Delamination (yellow-shaded) initiated at a discrete matrix crack. With an increasing number of cycles, the delaminated area grew in two directions, over the width of the weft (load direction) and along the weft direction (transverse to the load). With delamination reaching the area between the 0°  warp tows, accelerated longitudinal splitting commenced, followed by fiber bundle failure (FF) and specimen rupture. Again, no failure of the aluminum fibers could be ascertained in the in-situ micrographs.

Apart from the damage mechanisms, the microscope images also show that the aluminum fibers, which were straight and evenly spaced before autoclave curing, were bunched together and wavy afterwards. This is partly a consequence of the higher temperature expansion of the aluminum compared with the glass fibers. Waviness and, in particular, bundle formation also result from the relocation of the aluminum fibers into the spaces between the warp threads during autoclave processing. Improvements to the aluminum fiber distribution could be achieved in the future, if necessary, by selecting a finer glass fabric or a prepreg.

**Figure 19:**
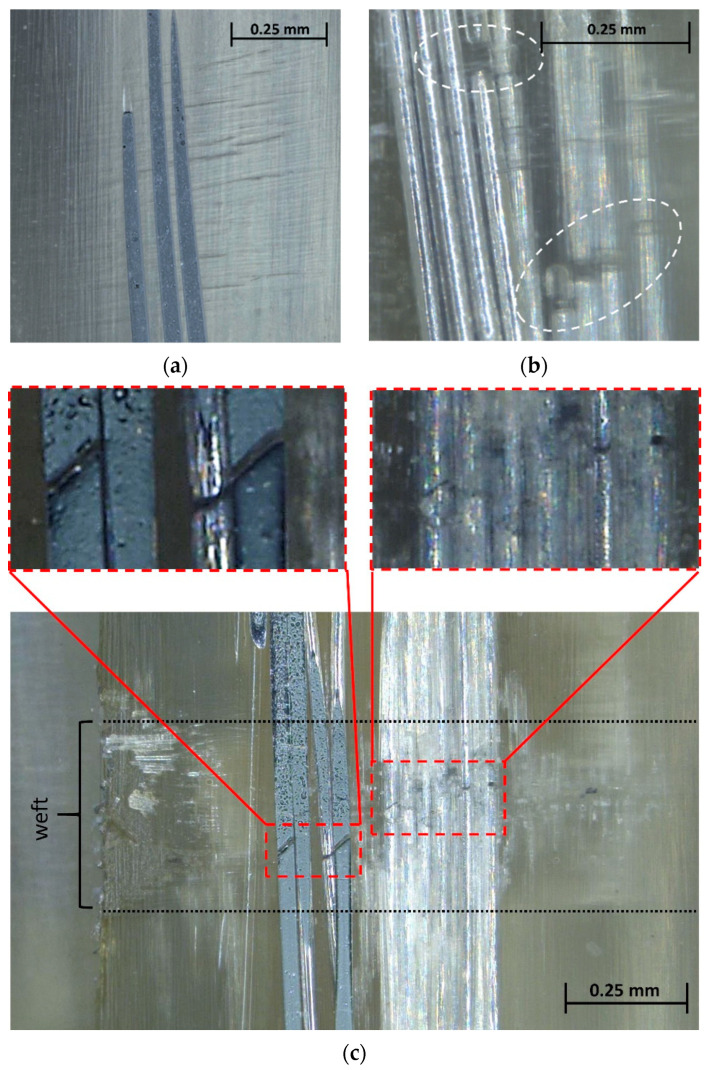
Post-rupture (offline) micrographs of the polished specimens showing inter fiber cracks (**a**) and internal aluminum fiber ruptures (**b**,**c**).

To further investigate the micro-damage features, additional post-failure microscopy was conducted for selected fatigue specimens. Sample micrographs are depicted in Figure 19. The micrograph Figure 19a shows the aluminum fibers exposed through mechanical preparation (grinding, polishing). The aluminum fibers appear to be undamaged; however, weft-internal inter-fiber cracks, as identified earlier by in situ microscopy (cf. Figure 18a–c), can be seen. In Figure 19b, apparent aluminum fiber ruptures can be seen. As the aluminum fiber bundle was not exposed by mechanical preparation and was still located below the polished surface, we can rule out the possibility that the damage was caused by the preparation of the micrograph. The overview image in Figure 19c again reveals that aluminum fiber ruptures primarily occurred in the vicinity of the crossing weft tow. Ruptures of aluminum fibers lying below the grinding plane (right detail picture) and ruptures of exposed aluminum fibers (left detail picture) can be seen.

Although thorough microscopy was conducted, only a relatively small number of aluminum fiber ruptures could be identified for the following potential reasons. Firstly, ruptures might have been difficult to identify due to the waviness of the aluminum fibers, their optical properties (high reflectance) and potential crack closure due to the plastic deformation of the aluminum. Secondly, the fatigue behavior of the aluminum fibers may have been better than anticipated due to, e.g., thermal softening associated with the autoclave processing, fiber waviness and supporting effects of the matrix enclosing the fibers. Thirdly, the fatigue strength of the GFRP phase might have been too similar to that of the aluminum fibers, so that they did not show pronounced failure, especially in the form of visible cracks. In fact, the fatigue performance of the GFRP laminate was very low (Section 4.2), and this can also be assumed to hold true for the GFRP phase in the AlFGFRP laminate. Overall, the fatigue life seems to crucially depend on the fatigue of the warp and weft tow crossings. While these crossings are known to be weak zones, high compaction forces during autoclave processing may have aggravated this condition, causing excessive local weakening. Unfortunately, neither SN curves of the GFRP material nor of the aluminum alloy, especially in the form of fine wires, could be found in the literature for comparison.

### 4.4. Electrical Resistance

The contacted AlFGFRP-2 (work-hardened aluminum fibers) specimens, as depicted in Figure 7c, were tested under static and fatigue loads to investigate the effect of mechanical loading on their electrical resistance. It should be mentioned at this juncture that the efforts made to manufacture this type of specimen were considerable. Hence, only 12 contacted specimens were available for all the resistance and electric load tests.

In the first step, the electrical resistance of a single aluminum fiber was characterized. Therefore, potentiostatic and potentiodynamic analyses of 280 mm long fiber segments were conducted to gain characteristic current versus voltage curves. The electric resistance was then calculated by means of Ohm’s law (potentiostatic data) and by means of linear regression analysis (potentiodynamic data), both giving identical resistances of 10.89 Ω. Taking the lengths and cross-section area into account, the average electrical resistivity was determined to be $6.1845\;\times 10^{-8}\;\Omega m$, which is in good agreement with values provided in the technical literature, for the EN AW-5019 alloy ($5.26\;\times 10^{-8}\;\Omega m$ to $6.67\;\times 10^{-8}\;\Omega m$).

**Figure 20:**
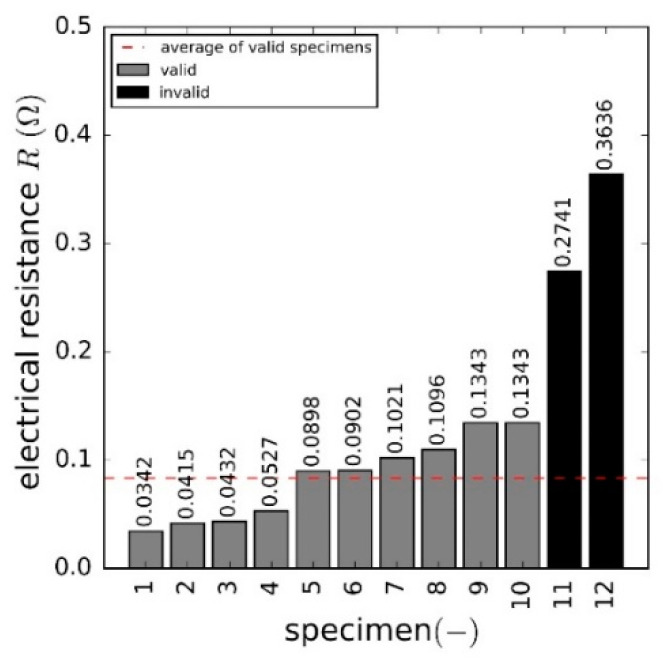
Electrical resistance of all the contacted specimens derived from potentiodynamic measurements.

In the second step, the electrical resistance of the unstressed contacted specimens was characterized. The results are summarized in Figure 20. Two specimens with an unusually high electrical resistance, presumably due to poor contacting of the aluminum fibers, were declared invalid and discarded. The absolute resistances of all other specimens ranged from 0.034 Ω to 0.134 Ω, with an average value of 0.083 Ω ±0.036 Ω. The high standard deviation was assumed to result from the varying contacting quality of the crimping connections. By comparison, the theoretical specimen resistance, considering an idealized parallel connection of approximately 1500 aluminum fibers with a length of 280 mm, resulted in a calculated electric specimen resistance of 0.0073 Ω plus about 0.00152 Ω for the AWG12 copper connector wires. Accordingly, the experimentally determined resistance was about ten times higher than the theoretical one, which was assumed to result from the crimping connections and influences, such as the surface oxidation of the aluminum and possible resin infiltration.

**Figure 21:**
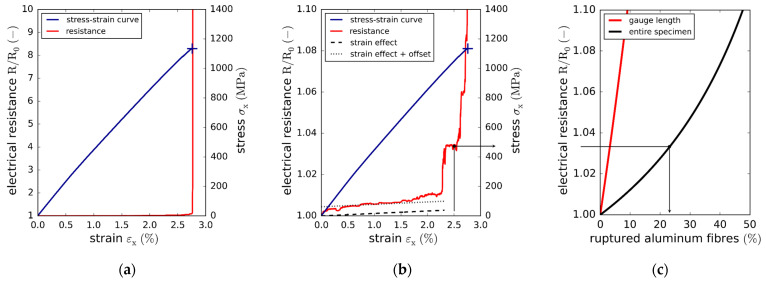
Exemplary resistance increase of a single specimen (potentiostatic measurements) during a quasi-static tension test (**a**), comparison with the estimated resistance increase due to strain (**b**), analytically calculated resistance increase due to the gradual rupture of the parallel non-touching aluminum fibers, according to Equation (7) (**c**).

Concerning the impact of quasi-static loads on the electrical resistance, Figure 21a,b depicts both the stress–strain curve and the changes in the normalized resistance derived from a continuous potentiostatic measurement (with a sampling frequency of 200 Hz). It can be seen that the resistance does not change significantly over a wide range of the longitudinal tensile strain until the catastrophic failure of the specimen at the ultimate strain (2.75%). Figure 21b reveals that, except for the steeper initial climb, the measured resistance increases nearly linearly until reaching a strain of 2.25%. This increase in the resistance is a direct consequence of the longitudinal strain, as can be shown by means of the simplistic model (Section 3.6). Substituting Equation (8) into Equation (7), and using the aluminum fiber properties (Poisson’s ratio, resistivity, length, diameter), the number of aluminum fibers per specimen yields the thick dashed straight line (strain effect). Shifting this curve upwards (strain effect plus offset) reveals that the slopes of both the experimental and analytically calculated resistance increases are in good agreement. This suggests that the majority of aluminum fibers remained intact over a wide range of strain.

Neglecting the strain-related resistance increase, the impact of the aluminum fiber ruptures on both the gauge length resistance and overall specimen resistance (including tabs and connection wires) is assessed by means of Equation (7) and plotted in Figure 21c. The unknown resistance of the crimp connection ($R_{\mathrm{CC}})\;$ was adjusted so that the analytical initial specimen resistance (Equation (7)) matched the initial experimentally measured value. The curve corresponding to the resistance of the entire specimen suggests that about 5% of the conductive fibers ruptured at $\varepsilon_{x}=2.0\%$. Shortly before the final failure, at $\varepsilon_{x}=2.5\%$, about 20% to 25% of the aluminum fibers ruptured, again assuming the validity of all the simplifications.

**Figure 22:**
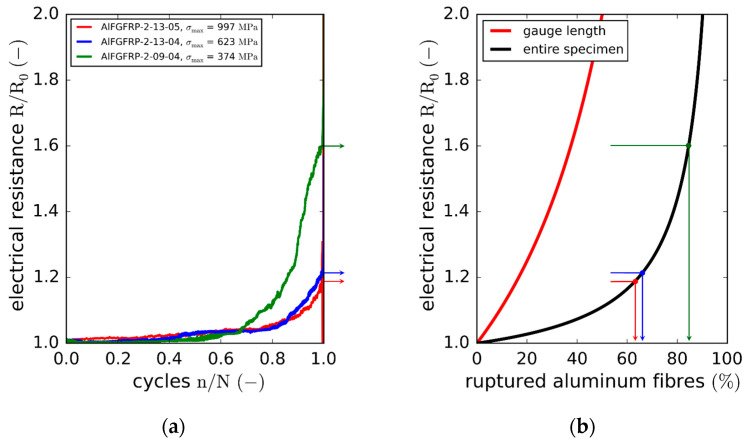
(**a**) Development of the electrical resistance of contacted AlFGFRP-2 specimens under fatigue loads; (**b**) analytically calculated resistance increases due to the gradual one-by-one ruptures of the parallel non-touching aluminum fibers, according to Equation (7).

While the specimen conductivity did not degrade significantly under quasi-static loads, fatigue loading was observed to have a more detrimental effect. Concerning the fatigue life, the average change of cycles to failure of AlFGFRP-2 (work-hardened aluminum) was only about −6.5% compared to the reference GFRP laminate. Microscopic investigations of the damage mechanisms revealed the occurrence of aluminum fiber ruptures; however, their identification was only possible post-rupture. The resultant normalized resistance curves (one specimen per load level) are plotted against the normalized fatigue life in Figure 22a. For the three load levels shown, the resistance remains nearly unchanged up to about 40% of the fatigue life. Then, a region of minor resistance increase can be observed up to 70% of the fatigue life. Afterwards, the resistance increases progressively for all three load levels. The resistance behaves similarly for the high and medium load levels, exhibiting increases of about 20% before the final failure. For the low load level, however, the resistance increase is more progressive and distinct. Before the loss of conductivity due to the final failure, the resistance increases to about 60%. Again, the simplified model in Equation (7) can be used to estimate the number of broken conductors (Figure 22b). In the case of the high and medium load levels, the model yields a fraction of interrupted aluminum fibers of about 65%, while for the low load level, about 85% of the aluminum fibers are assumed to have failed due to the fatigue loading.

### 4.5. Electrical Load

**Figure 23:**
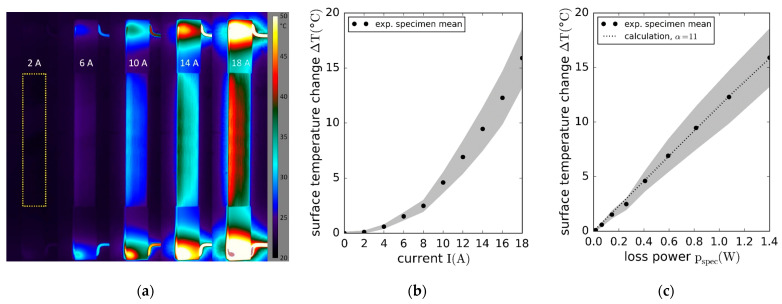
(**a**) Thermographic pictures of a current-carrying specimen; (**b**) measured surface relative temperature (gauge section mean temperature) plotted against the current; (**c**) measured and calculated temperature plotted against the gauge section loss power.

For one specimen, electrical load tests were conducted to investigate the current-carrying capacity and heating behavior of the contacted AlFGFRP-2 specimens. Electrical currents of up to 26 A were applied, using the electrical circuit shown in Figure 9a, with $R_{S}$ representing the total ohmic resistance of the contacted specimen (Equation (7)). The supply voltage, as well as the system resistance, were adjusted to obtain the desired current passing through the specimen. Thermographic imaging was employed for the temperature monitoring. Figure 23a shows sample images of a specimen carrying currents ranging between 2 A and 18 A. The images reveal the imperfect contacting of the aluminum fibers, indicated by local heating in the end tab regions. End tab heating was found to be more critical than the heating of the gauge section laminate. At a current of 26 A, local heating caused desoldering of the copper wires. Considering the heating behavior in the gauge section, some specimens showed a non-uniform temperature distribution. In the case of the sample specimen (Figure 23a), a temperature difference of about 5 °C can be seen between the left and right specimen edges. With the aluminum fiber distribution being relatively homogeneous (except for the local waviness), non-uniform heating indicates a non-uniform current distribution. This most likely stems from non-uniform crimping connections in combination with missing (or lower) transverse interconnection of the conductors compared to a conventional cable wire.

The equilibrium temperature in the relative mean gauge section (yellow box) is plotted against the electric current in Figure 23b, suggesting a quadratic relationship. The grey-shaded region represents the standard deviation of the temperature in the gauge section. Additionally, the temperature increase was calculated iteratively by means of Equation (10), starting with the theoretical gauge section resistance at room temperature. The temperature increases linearly with the loss power in the gauge section. The experimental temperatures and calculations correlate well for a thermal heat transfer coefficient $\alpha =11\;W/\left(m^{2}K\right)$ and passive convection (literature: 5≤α≤15).

The investigation of the ohmic heating behavior also helped to identify a suitable current for the combined electro-mechanical tests. With both the ohmic heating and autonomous heating contributing to an increase in the specimen temperature, the current should be controlled carefully in order to prevent specimen overheating. A current of 10 A  was identified to be suitable for all the specimens and load levels.

### 4.6. Combined Mechanical and Electrical Load

Similar to the investigations of the electrical resistance (Section 4.4), quasi-static and fatigue tests were conducted using the electrically contacted specimens (AlFGFRP-2). However, this time, a pre-set direct current was applied to investigate the effect of the electrical load on the specimens’ mechanical behavior. The voltage was kept below 12 V for safety reasons. The test set-up depicted in Figure 9 was used to apply the electrical loads. With the specimen resistance being much lower than the system resistance, damage-related current decreases were low and could easily be compensated by manually increasing the supply voltage. Thus, the tests were conducted using a quasi-constant current.

**Figure 24:**
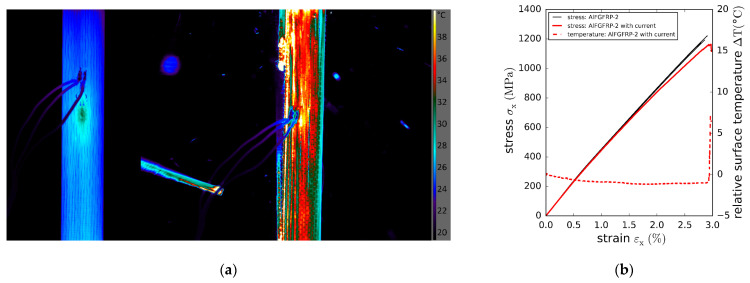
Combined tensile test with electrical load: (**a**) thermographic images of a mechanically unloaded specimen carrying a current of 5 A (left) and at the moment of final rupture (right); (**b**) stress–strain results and surface relative temperature of the current- and non-current carrying specimen.

Due to the small number of available contacted specimens, only a single quasi-static test was conducted. The results are depicted in Figure 24. Figure 24a shows the surface temperature distribution at a current of 5 A without mechanical load (left), and at the moment of the catastrophic specimen rupture (right). The left image reveals that the specimen temperature distribution is rather homogeneous, except for slightly elevated temperatures in the vicinity of the strain gauge. The initial average surface temperature is 26.8 °C. Along with the increasing strain (Figure 24b), a slight decrease in the surface temperature can be observed. The cause of this behavior could not be ascertained. With the ambient temperature being constant throughout the test, it might be a result of strain-related changes in the heat transfer or heat emissivity. A steep increase in temperature can be seen at the moment of specimen rupture. This is in accordance with the measured abrupt increase in the specimen resistance and is also a result of fracture surfaces, revealing the specimen’s core temperature. The comparison of the stress–strain curves of the current-carrying and solely mechanically loaded specimens reveals slight differences. The stress–strain curve of the current-carrying specimen becomes more non-linear at strains above 1.5%, and the strain at rupture $\varepsilon_{x,f}^{\mathrm{AlFGFRP}\text{-}2,\mathrm{curr}}=2.97\%$ is slightly higher compared to $\varepsilon_{x,f}^{\mathrm{AlFGFRP}\text{-}2}=2.85\%$, which is assumed to be a consequence of the specimen’s temperature. In contrast, hardly any impact on the tensile strengths ($\sigma_{x,f}^{\mathrm{AlFGFRP}\text{-}2,\mathrm{curr}}=1163\;\mathrm{MPa}$ compared to $\sigma_{x,f}^{\mathrm{AlFGFRP}\text{-}2}=1197\;\mathrm{MPa})$ was observed.

**Figure 25:**
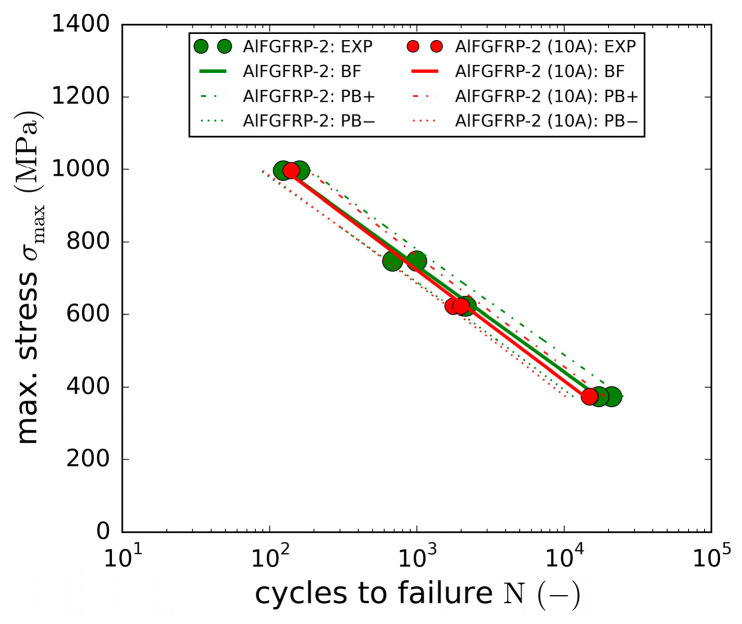
SN curves of AlFGFRP-2 with regression curves and prediction bands: sole mechanical fatigue loads (green) and combined electro-mechanical loading with a constant current of 10 A (red).

As the electric specimen resistance undergoes a more distinct degradation under cyclic loads, we set our focus on combined electric and fatigue testing (R=0.1). In this unique test series, five contacted specimens were tested at three load levels, according to Section 4.2. The results in terms of the SN curves are plotted in Figure 25, along with the previous results from Figure 13a. A comparison of the SN curves reveals a rising difference towards lower load levels. While there is hardly any influence on the fatigue life at the high load level (−1.4%), the cycles to failure changed by about −12% at the medium load level. At the low load level, the fatigue life changed by −22% compared to the solely mechanically loaded specimen.

**Figure 26:**
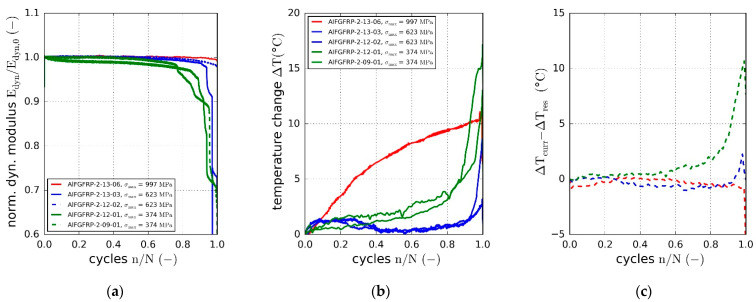
Test results of the combined electric and mechanical fatigue load testing: dynamic stiffness (**a**), surface temperature changes (**b**) and temperature differences between the electrically loaded and unloaded specimens (**c**).

The investigation of the stiffness degradation (Figure 26a) and specimen heating (Figure 26b,c) revealed further differences. With the stiffness being constant over large parts of the fatigue life, overall degradation behavior was similar to that of the sole fatigue load. This was true for the high and the medium load levels. However, for the low load level, the stiffness degradation was much more distinct during the last 20% of the fatigue life. In fact, both specimens tested at the low load level exhibited a gradual loss of stiffness between 80% and 90% of the cycles to failure. Then, during the last 5% of the fatigue life, the stiffness dropped suddenly to 70% of the initial value. Stiffness reductions were observed to occur simultaneously with longitudinal splitting. This damage mechanism was also observed under sole mechanical loading (shortly before final failure); however, it seemed to occur earlier under combined mechanical and electrical loads. In consequence, the final specimen rupture was less abrupt, showing less distinctive specimen disintegration.

Similar to the stiffness degradation results, hardly any differences in the heating behavior could be seen at the high and the medium load level. Figure 26b shows the change in the average surface temperature over the normalized number of cycles. Additionally, Figure 26c depicts the temperature differences between specimens tested under mechanical and combined electrical-mechanical loads. While the temperature differences are negligible at the high and the medium load levels, the specimen heating is more distinct at the low load level. In fact, with the electric load, the average surface temperatures rose up to 10 °C  higher before the final failure.

**Figure 27:**
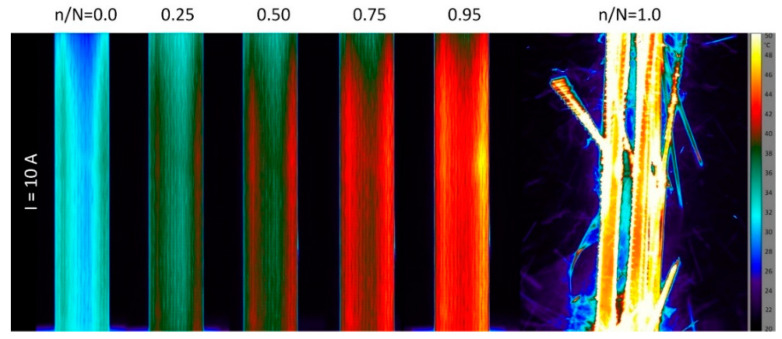
Thermographic image series of a current-carrying specimen (10 A) under a high fatigue load (997 MPa).

**Figure 28:**
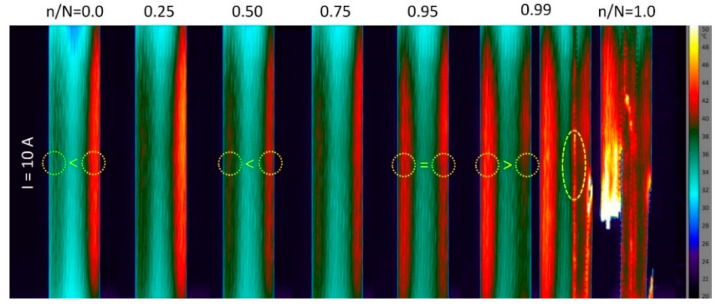
Thermographic image series of a current-carrying specimen (10 A) under a low fatigue load (374 MPa), showing inhomogeneous ohmic heating, high temperature zone shifting and longitudinal splitting.

While the surface temperature distributions were rather homogeneous under the solely mechanical fatigue loads, distinct temperature gradients were observed under the electric loads (Figure 23) and also under the combined electrical-mechanical loads. Figure 27 shows a series of thermographic images taken during fatigue testing at the high load level. The first image shows the temperature distribution caused only by the electric load. The image reveals two roughly 8 mm-broad bands near the free edges, which are about 6 °C warmer (36 °C) than the specimen middle region (30 °C). This inhomogeneous distribution of the surface temperature persists throughout the fatigue life, even though the overall specimen temperature increases due to the autonomous and damage-related heating. The resulting temperatures of around 40 °C (at 75% of the cycles to failure) are higher, as desired; however, they are sufficiently below the glass transition temperature of the hot curing epoxy system. An interesting phenomenon can be observed in Figure 28, showing the temperature distributions at the low load level (374 MPa) and 10 A current. Again, the initial temperature distribution due to ohmic heating is inhomogeneous. Additionally, with a difference of 6 °C between the edge bands (the right band is warmer), the temperature distribution is not symmetric. Strain- and damage-related heating plays a subordinate role at this load level, and no significant rise in the overall specimen temperature can be seen throughout the fatigue life (compared to the results in Figure 16b). However, over the specimen life cycle, an interesting shift in the high temperature zones can be seen. While the right edge band is warmer in the beginning, a decrease in temperature can be seen from n/N=0.25 (45 °C) to n/N=0.5 (41 °C). Simultaneously, the temperature of the left band increases (to 39 °C) compared to the initial state (n/N=0, 36 °C). At n/N=0.95, both bands show a similar temperature of about 42 °C. Then, shortly before the final failure at n/N=0.99, the right band abruptly cools down to below 40 °C, and longitudinal splitting (elliptic marker) along the former left boundary of the temperature band occurs, followed by the catastrophic specimen rupture. With the accumulation of damage and, especially, with the occurrence of local plasticity and aluminum fiber ruptures being unknown, an explanation for the temperature shift phenomenon is speculative. As mentioned before, the inhomogeneity of the initial temperature redistribution is most likely caused by the improper contacting of the aluminum fibers. Hotter regions indicate higher local currents due to a better contacting/lower resistance. Likewise, local decreases in the temperature are most likely caused by local damage-induced resistance increases. As a consequence, the current increases in the intact or less damaged part of the cross-section. This explanation for the high temperature zone shift is supported by the fact that the final failure-inducing longitudinal splitting occurs directly along the cooled-down edge band.

### 4.7. Comparison to Conventional Aluminum Cables

Typical aluminum cables, such as those used in automotive applications, are made from 1000 series aluminum alloy, such as EN AW-1370 or similar, with an electrical conductivity of about 62% of copper or a maximum resistivity (soft condition) of about $2.8\;\times \;10^{-8}\;\Omega m\;$[42,43,44,45]. The experimentally determined resistivity of EN AW-5019 alloy fibers is about 120%  lower ($6.1845\;\times 10^{-8}\;\Omega m$) (Section 4.4). However, their tensile strength is about five times higher (511 MPa) (Table 3). As stated above (Section 4.4), the experimentally obtained electric resistivities of the contacted specimens were much lower than the analytical estimations due to the contacting issues.

**Figure 29:**
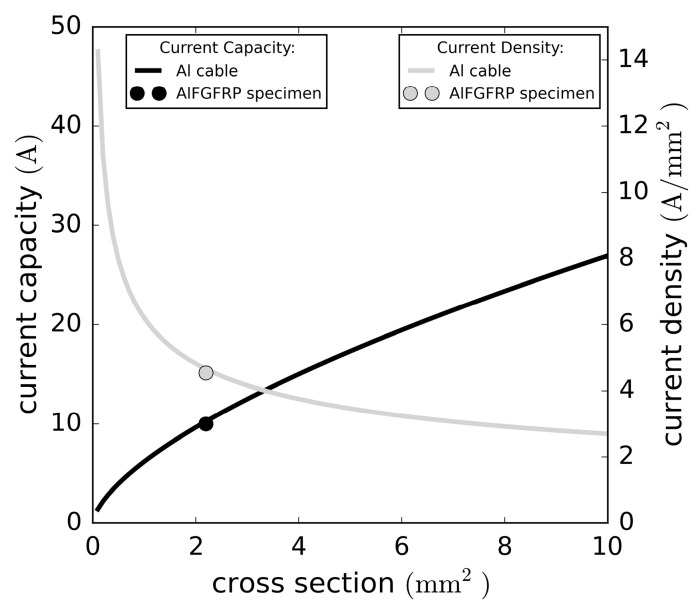
Current ratings of conventional single-core aluminum cables based on [46], with data points below 4 mm^2^ resulting from extrapolation: comparison with the current load of the aluminum phase of the AlFGFRP-material.

The current rating of conventional aluminum cables depends primarily on the conductor diameter. For a comparison with the multifunctional material, a regression analysis of the current rating data of single-core-XLPE-insulated aluminum cables [46] and an extrapolation to cross-sections smaller than $4\;{\mathrm{mm}}^{2}$ was conducted. The current ratings and resulting current densities are plotted against the cross-section in Figure 29. The effective aluminum cross-section of the AlFGFRP specimens was about $2.4\;{\mathrm{mm}}^{2}$. With the current set to 10 A in the combined electrical-mechanical tests, and a resulting current density in the aluminum of about $4.5\;A/{\mathrm{mm}}^{2}$, the current load was similar to the current capacity of a conventional cable. Considering the full multifunctional material, the average current density was about $\;320\;\mathrm{mA}/{\mathrm{mm}}^{2}$.

## 5. Discussion

The main objective of this work was to ascertain whether a multifunctional material can carry technically relevant electrical currents without its mechanical properties being affected. This requirement is of fundamental importance with respect to technical applications. Although several studies have investigated multifunctional composites containing fibrous metals, knowledge of fibrous aluminum-based composites and, especially, of their behavior under combined electrical-mechanical loads is rare to non-existent.

Combined electrical-mechanical fatigue tests, including microscopy, potentiometry and thermography, were conducted to investigate the electrical, mechanical and combined performance of a multifunctional aluminum-fiber-reinforced GFRP material containing 10 vol.%  of work-hardened aluminum fibers with a diameter of 0.045 mm. The results of the combined tests indicate that there were no significant fatigue life reductions at high and medium load levels when carrying a current of 10 A (current density of 4.5 A/mm^2^ in the aluminum), which corresponds to the current rating of conventional aluminum cables. Consequently, the current itself does not affect the mechanical behavior. A notable reduction in the fatigue life (−20%) at the low load level (30%UTS) can be attributed to the ohmic heating of the specimen. In fact, temperature is known to be an important variable influencing the fatigue behavior [37,38]. Additionally, temperature induced stresses due to the non-matching thermal expansion coefficients of aluminum and glass fibers may also have detrimental effects; however, this requires further investigation beyond the scope of the present work. While the lack of compatibility of the thermal expansion coefficients cannot be adjusted for the given constituents, the ohmic specimen heating can be improved by better electrical contacting and the realization of a more homogeneous temperature distribution.

Under sole quasi-static tension loading, the resistance of the specimens remained almost unchanged until specimen rupture. Thereby, the resistance increase due to mechanical strain was negligible, which has also been reported by Pototzky [23] for different sheet metals. The observation that the electrical resistance did not undergo notable changes, even at strains greater than the rupture strain of the free single aluminum wire, could not be clarified. It stands to reason that the rupture strain of the fibers increased due to thermal softening (during autoclave processing), which is a known phenomenon of work-hardened AlMg alloys [47]. Further reasons for the observed behavior may be the waviness of the aluminum fibers and their cross-contacting. Positive effects of thermal residual stresses are unlikely, since these would equate to tensile stresses in the aluminum. Wang et al. [48], for example, reported a reduction in the yield point of GLARE caused by thermal residual tensile stresses in the aluminum. At high, medium and low fatigue load levels, the electrical resistance increased progressively in the last 30% of the fatigue life, which is a consequence of aluminum fiber ruptures. It is known that plastic deformation also leads to resistance increases, but only to a small extent [49].

A comparison with the experimental results of the GFRP reference material reveals that metal fiber integration affected neither the fiber-parallel tensile strength nor the fatigue strength significantly. Similar results were reported by Hannemann et al. [50] for a steel-fiber-reinforced CFRP material under quasi-static loading. In a later publication, it was also shown that the fatigue life was not significantly affected [51]. In contrast, the static transverse strength decreased significantly (−17%) due to the inadequate bonding of the aluminum fibers to the polymer matrix. The bonding of aluminum requires a thorough physical and chemical surface treatment due to the formation of weak natural oxide layers [52,53]. Therefore, the inline surface preparation of the thin metal fibers should be improved as far as possible, for example, by including a chemical surface treatment. Nevertheless, aluminum bond strength apparently was sufficient to not cause significant fatigue life reductions compared to the reference laminate. The results also demonstrate the importance of geometrical and mechanical compatibility. Concerning the geometry of the aluminum fibers, the integration of thinner fibers led to a higher transverse tensile strength. In contrast to the initially work-hardened wire, the soft-annealed wire was less compatible with the stress–strain behavior of the glass fibers. The plastic deformation of the aluminum, indicated by higher hysteretic losses, led to the lower static and fatigue strength of the composite.

Our important finding that the integration of work-hardened aluminum did not cause significant fatigue life reductions must be taken with caution, however, as the fatigue strength of the GFRP base laminate was comparatively low (c.p. [54]). The latter requires verification for the specific laminate fabrication processes used herein in contrast with other GFRP materials. Of course, the same holds true for the impact of the electrical current on the fatigue behavior.

From a technical point of view, the contacting of thin aluminum fibers is a central problem to be solved, especially in the case of finer fiber integration approaches, such as homogeneous hybrid single plies. This is equally true for relatively simple test specimens and for contacting solutions for the power and signal transfer in multifunctional lightweight structures [23,55]. The improvement of the contacting, along with the improvement of the fiber–matrix bonding, is thus central task for further investigations.

Overall, the present pioneering experimental results of the mechanical and electrical properties appear to be promising, especially in view of the non-ideal material architecture and specimen fabrication. Discounting the undesired temperature influence, the multifunctional material is capable of carrying technically relevant currents. In contrast to steel-based composites, the integration of aluminum fibers into GFRP does not cause an increase in density. Mass savings through the elimination of conventional cables depend significantly on the application and even more on the required cable cross-sections. Therefore, mass savings cannot be estimated indiscriminately nor on the basis of this initial research.

## 6. Conclusions

A multifunctional aluminum fiber-glass-fiber-reinforced polymer (AlFGFRP) proposed as a replacement for conventional electric conductors in future electric vehicles was investigated experimentally to assess its mechanical, electrical and combined material behaviors. Sophisticated monitoring methods (potentiometry, thermography, microscopy) were applied to investigate the damage of the composite and, especially, the conductive aluminum reinforcement.

The main conclusions of this work are as follows:

- The integration of a small amount (10 vol.%) of work-hardened aluminum fibers into a unidirectional GFRP material does not significantly affect the tensile strength and fatigue strength in the fiber-parallel direction.
- The transverse strength is reduced significantly (−17%) due to the inadequate bonding of the aluminum fibers, revealing the need for improved surface treatments.
- The electrical conductivity is widely maintained until the specimen rupture under static loads, whereas it decreases under cyclic loading within the last third of the fatigue life due to the fatigue of the aluminum fibers.
- Technical relevant currents (320 mA/mm^2^) can be carried; however, the fatigue life is reduced at low load levels due to temperature effects stemming from the inhomogeneous ohmic heating.
- The contacting of thin aluminum fibers is a central problem to be solved, especially in the case of finer fiber integration approaches, such as homogeneous hybrid single plies.

In addition to solving the aluminum fiber contacting and bonding, there are several fundamental questions that need to be answered in future investigations. As a first step, due to the limited number of specimens tested and the unusual low fatigue strength of the base GFRP composite, further GFRP materials and more homogeneous aluminum fiber distributions should be investigated. Thus far, proof of the current-carrying capability is limited to low voltages. However, for many technical applications, such as electric propulsion, a high-voltage energy transfer is more appropriate. Therefore, the impacts of both high currents and high voltages should be studied. The damage behavior is assumed to be more complex at high voltages due to potential arcing at the fiber interruptions and/or between plies. Future engineering challenges will involve aspects of industrial fabrication, the design of durable and reliable contactors and their integration in electrical systems, not forgetting the numerous questions concerning safety and repairability.

## Figures and Tables

**Table 1:** Typical mechanical and electrical properties of aluminum (pure/alloys), E-glass and epoxy matrix [30,31,32,33].

Property	Unit	Aluminum	E-Glass	Epoxy Matrix
Elastic modulus	GPa	68–72	50–80	2.75–4.1
Shear modulus	GPa	25–28	20–25	1.2–1.5
Poisson’s ratio	-	0.35	0.2	0.2–0.35
Yield strength	MPa	17–480	-	-
Yield strain	%	0.03–0.7	-	-
Tensile strength	MPa	45–538	3450–3790	40–90
Max. tensile strain	%	2–60	3–4.8	3–7
CTE *	1/K	25 × 10^−6^	5 × 10^−6^	50 × 10^−6^–80 × 10^−6^
Electrical resistivity	Ωm	2.82 × 10^−8^–8.2 × 10^−8^	10^15^	>10^10^
Density	g/cm^3^	2.6–2.7	2.54–2.6	1.2–1.3

* Coefficient of thermal expansion.

**Table 2:** Properties of the constituent materials.

Property	Unit	E-Glass Fiber	Carbon Fiber	Aluminum	Steel	Epoxy
$\mathrm{Elastic}\;\mathrm{modulus}\;\mathrm{in}\;\mathrm{fiber}\;\mathrm{direction},\;E_{1,i}$	GPa	78,000	240,000	70,000	176,000	3300
$\mathrm{Elastic}\;\mathrm{modulus}\;\mathrm{transverse}\;\mathrm{to}\;\mathrm{fibers},\;E_{2,i\;}$	GPa	78,000	24,000	70,000	176,000	3300
Poisson’s ratio, υ	-	-	-	-	-	0.38
$\mathrm{Electrical}\;\mathrm{resistivity},\;r_{1,i}$	Ωm	$1\;\times 10^{14}$	$1.6\;\times 10^{-5}$	$5.26\;\times 10^{-8}$	$6.97\;\times 10^{-7}$	$1\;\times 10^{13}$
$\mathrm{Density},\;\rho_{i}$	g/cm^3^	2.6	1.77	2.64	7.95	1.2

**Table 3:** Properties of the EN AW-5019 wires after heat treatment (180 °C, 3 h).

As-Supplied Condition	Yield Strength MPa	Tensile Strength MPa	Elastic Limit %	Failure Strain %
Cold-worked	-	511	0.62	0.81
Soft-annealed	164	279	0.19	8.92

**Table 4:** Results of the longitudinal tensile tests.

Material Configuration	Thickness t/mm	Width w/mm	Modulus $E_{x}/mm$	Strength $\sigma_{x,f}/MPa\;$	Failure Strain $\varepsilon_{x,f}/\%$
GFRP	1.12 ± 0.020	25.02 ± 0.01	47,211 ± 640	1246 ± 18	2.76 ± 0.02
AlFGFRP-2	1.26 ± 0.020	25.05 ± 0.01	47,735 ± 253	1197 ± 19	2.85 ± 0.05
AlFGFRP-3	1.25 ± 0.003	25.05 ± 0.03	43,279 ± 86	1124 ± 18	2.75 ± 0.04

**Table 5:** Results of the transverse tensile tests.

Material Configuration	Thickness t/mm	Width w/mm	Modulus $E_{y}/mm$	Strength $\sigma_{y,f}/MPa$	Failure Strain $\varepsilon_{y,f}/\%$
GFRP	1.13 ± 0.02	25.05 ± 0.02	19,489 ± 449	94.6 ± 2.19	0.56 ± 0.005
AlFGFRP-1	1.36 ± 0.002	25.08 ± 0.02	16,820 ± 435	73.8 ± 0.9	0.52 ± 0.03
AlFGFRP-3	1.30 ± 0.01	25.05 ± 0.03	17,609 ± 294	78.61 ± 1.34	0.52 ± 0.01

## Data Availability

Data supporting the findings of this study are available within the article and upon request from the corresponding author.
